# Neutral and Acidic Oligosaccharides Supplementation Does Not Increase the Vaccine Antibody Response in Preterm Infants in a Randomized Clinical Trial

**DOI:** 10.1371/journal.pone.0070904

**Published:** 2013-08-08

**Authors:** Jolice P. van den Berg, Elisabeth A. M. Westerbeek, Fiona R. M. van der Klis, Guy A. M. Berbers, Harrie N. Lafeber, Ruurd M. van Elburg

**Affiliations:** 1 Department of Paediatrics, Division of Neonatology, VU University Medical Center, Amsterdam, The Netherlands; 2 Laboratory for Infectious Diseases and Screening, National Institute of Public Health and the Environment, Bilthoven, The Netherlands; 3 Centre for Specialised Nutrition Danone Research Wageningen, The Netherlands; Glaxo Smith Kline, Denmark

## Abstract

**Background:**

In preterm infants, a decreased immunological response and lower serological effectiveness are observed after immunizations due to ineffectiveness of both humoral and cellular immune mechanisms.

**Objective:**

To determine the effect of 80% neutral oligosaccharides [small-chain galacto-oligosaccharides/long-chain fructo-oligosaccharides (scGOS/lcFOS)] in combination with 20% pectin-derived acidic oligosaccharides (pAOS) on antibody concentrations after DTaP-IPV-Hib immunization in preterm infants.

**Design:**

In this randomized clinical trial, preterm infants with gestational age <32 weeks and/or birth weight <1500 g received enteral supplementation with scGOS/lcFOS/pAOS or placebo (maltodextrin) between days 3 and 30 of life. Blood samples were collected at 5 and 12 months of age.

**Results:**

In total, 113 infants were included. Baseline and nutritional characteristics were not different in both groups. Geometric mean titers were not different after prebiotic supplementation at 5 months, Ptx (37/44 EU/ml), FHA (78/96 EU/ml), Prn (78/80 EU/ml), Diphtheria (0.40/0.57 IU/ml), Tetanus (0.74/0.99 IU/ml) and Hib (0.35/0.63 µg/ml), and at 12 months Ptx (55/66 EU/ml), FHA (122/119 EU/ml), Prn (116/106 Eu/ml), Diphtheria (0.88/1.11 IU/ml), Tetanus (1.64/1.79 IU/ml) and Hib (2.91/2.55 µg/ml).

**Conclusions:**

Enteral supplementation of neutral (scGOS/lcFOS) and acidic oligosaccharides (pAOS) does not improve the immunization response in preterm infants.

**Trial Registration:**

Controlled-Trials.com ISRCTN16211826 ISRCTN16211826

## Introduction

Preterm infants have an immature immune system. Moreover, preterm infants receive less IgG from their mothers during pregnancy than term infants, which makes them vulnerable for infectious diseases during the first months of life. [1], [2], [3] Primary immunizations of infants in the Netherlands with a diphtheria, tetanus, acellullar pertussis, polio and *Haemophilus influenzae type b* combination vaccine (DTaP-IPV-Hib) are recommended at the age of 2, 3 and 4 months followed by a booster dose at 11 months, irrespective of gestational age (GA) at birth [4].

In preterm infants, a decreased immunological response and lower serological effectiveness are observed after immunizations due to ineffectiveness of both humoral and cellular immune mechanisms. These have been measured in antibody responses after vaccinations. Lower antibody responses to Hib have been found in several studies in preterm infants with a GA <32 weeks compared with term infants. [5], [6], [7] Moreover, immunoglobulin G (IgG) antibody response after acellullar pertussis immunization was lower in preterm infants compared with term infants [8].

There is accumulating evidence that intestinal microbiota have an important role in the postnatal maturation of the immune system. Intestinal bacteria play a key role in promoting the early development of the intestinal mucosal immune system both in terms of its physical components and its function, and continue to have a role later in life. [9], [10] The first month after birth is an important period in the development of the immune system. Singhal et al. emphasize that early nutrition has long-term health effects and the first month of life is a critical window for this effect. [11] Human milk stimulates the development of a bifidogenic intestinal microbiota. It is suggested that human milk, besides providing passive protection via immunoglobulins and other factors, plays an active role in the development of the infant immune system. [12] Human milk contains oligosaccharides which have immunomodulatory, anti-adhesive, and antimicrobial effects. [13] Over 200 human milk oligosaccharides have been identified with significant variability between individuals over time. [14] Of human milk oligosaccharides, 80% are neutral and up to 20% are acidic. In previous studies in preterm and term infants, supplementation with neutral oligosaccharides stimulated a bifidogenic intestinal microflora with a decrease of pathogens. [10], [15], [16] Newborn infants have an immune system which is skewed towards a Th-2 profile. [17] Human milk oligosaccharides have been shown to influence the modulation of the balance of Th1/Th2 immunity. [13], [18], [19] Neutral non-human milk oligosaccharides, such as small-chain galacto-oligosaccharides (scGOS) and long-chain fructo-oligosaccharides (lcFOS) have been developed to substitute these beneficial effects of human milk oligosaccharides [20], [21], [22], [23].

Non-human pectin-derived acidic oligosaccharides (pAOS) are able to act as receptors-analogs and are known to inhibit the adhesion of pathogens on the epithelial surface. pAOS may also directly affect the immune cells via interaction of selectins, dendritic cell specific C-type lectin, integrins, and other target receptors as Toll-like receptors. [15] In mice, fructo-oligosaccharides are able to improve the immune response to oral vaccination against Salmonella vaccine [24] and Vos et al. [25] described that the combination of scGOS/lcFOS (9∶1) and pAOS enhances vaccine-specific delayed type hyper-sensitivity responses in a dose-dependent manner. Furthermore, this was accompanied by a reduction of T-helper 2 cytokines production which indicates a Th1 skewed systemic immune response to the vaccine. This effect exerts especially during the early phase of the vaccine response [25].

As a result of these effects, we hypothesize that preterm infants who receive a combination of scGOS/lcFOS/pAOS may have an improved immunization response, reflected by higher levels of specific antibodies after their primary and booster DTaP-IPV-Hib immunizations. Therefore, the aim of this study was to measure the effect of enteral supplementation of neutral and acidic oligosaccharides during day 3 to day 30 of life on the vaccine response in preterm infants. [26].

## Materials and Methods

### Study Population

Infants born between May 2007 and October 2008 with a GA <32 weeks and/or birth weight (BW) <1500 g who were admitted to the level III neonatal intensive care unit (NICU) of the VU University Medical Center (VUmc) of Amsterdam, were eligible for participation in the study. Exclusion criteria were small for gestational age, infants with a GA >34 weeks (even with BW <1500 g), major congenital or chromosomal anomalies, death <48 hours after birth and transfer to another hospital <48 hours after birth.

### Ethics Statement

This study was part of the CARROT study and conducted according to the guidelines laid down in the Declaration of Helsinki and the medical ethical review board of VU University Medical Center approved all procedures involving human patients. Written informed consent was obtained from all parents. This trial was registered at isrctn.org as ISRCTN16211826. The protocol for this trial and supporting CONSORT checklist are available as supporting information; see Checklist S1 and Protocol S1.

### Immunization Schedule

All infants were scheduled to receive 4 doses of DTaP-IPV-Hib vaccine (Infanrix®, GlaxoSmithKline or Pediacel®, Sanofi Pasteur MSD) simultaneously administered with pneumococcal vaccine (Prevenar®, Wyeth) at 2, 3, 4 and 11 months of age following the Dutch national immunization programme. Infants also received HepB vaccine included in the hexavalent DTaP-IPV-Hib-Hep vaccine (Infanrix hexa®, GlaxoSmithKline) when one of their parents was born in a hepatitic endemic area. Immunizations were administered in the routine manner in the hospital or at the well baby clinic. The exact timing of the immunizations of every infant was recorded. Parents were asked to fill in a questionnaire with ten different questions on adverse experiences following infant vaccinations as previously described [27].

### Nutritional Support

Protocol guidelines for the introduction of parenteral and enteral nutrition followed current practices at our NICU. Nutritional support was administered as previously described. [28] In short, the medical staff of our NICU had final responsibility for the administration of parenteral nutrition and advancement of enteral nutrition. During the study period infants received from day 3–30 of life scGOS/lcFOS/pAOS or placebo (maltrodextrin) supplemented in increasing dose to a maximum of 1.5 g/kg/d to breast milk or preterm formula. Researchers, care providers and parents of participants were blinded for the given intervention. When the infant was transferred to another hospital the protocol was continued. After discharge, all infants received breast milk or preterm formula (Nenatal Start without oligosaccharides, Nutricia, Zoetermeer) until 3500 g and a postdischarge formula (Nenatal 1 without oligosaccharides, Nutricia, Zoetermeer) until the corrected age of 6 months.

### Laboratory Analyses

Blood samples were collected within 48 h after birth (t = 0, birth), 4–6 weeks after the third DTaP-IPV-Hib immunization (t = 1, 5 months) and 4–8 weeks after the fourth (booster) immunization (t = 2, 12 months). Serum samples were centrifuged and stored at –80°C until analysis. Serum samples were analyzed as previously described by van Gageldonk et al. [29] In short, serum samples were tested for IgG antibodies to pertussis toxin (Ptx), filamentous hemagglutinin (FHA), pertactin (Prn), diphtheria toxin (Dtx) and tetanus toxin (Ttx) with a multiplex immuno assay (Bio-Rad Laboratories, Hercules, CA) using Luminex technology. Serum samples were also tested for antibodies to Hib in a comparable immunoassay [30].

### Data Analyses

The sample size of 113 infants was based on the sample size calculation for the primary outcome of the main trial (the effect of scGOS/lcFOS/pAOS supplementation on serious infectious morbidity). [28] This was based on the difference in incidence in infectious morbidity (76% in the placebo and 50% in the intervention group, respectively) in a previous study. [31] Normally distributed and nonparametric data were presented as means (± SD) and median (range), respectively. For statistical analyses, antibody levels below the lower limit of quantitation were assigned as half the lower limit of quantitation (0.0005 IU/mL for Dtx and Ttx, 0.005 ug/ml Hib, 0.5 EU/ml for Ptx, FHA, Prn and Fim). All IgG antibody levels were expressed as geometric mean concentrations (GMCs) with 95% confidence intervals (CIs). Internationally assigned protective concentrations were used to determine the percentage of infants with protective IgG levels (anti-Dtx and anti-Ttx≥0.1 IU/ml, anti-Hib≥1 µg/ml). [32], [33], [34] Protective concentrations for anti-Ptx are not internationally assigned, but an arbitrary cut-off value of >20 EU/ml for protection was used as described previously. [35] Patient and nutritional characteristics were analysed with Student’s t-test, chi-square test or Fisher’s exact test for continuous normally distributed and dichotomous data, respectively. If parameters had a skewed distribution, a natural logarithmic transformation was performed before analysis. The effect of scGOS/lcFOS/pAOS on the antibody concentrations at 5 and 12 months were analysed with Student’s t-test. An additional linear regression analysis was performed to determine the influence of GA, BW, cord blood antibody concentrations, breastfeeding, infections during the first 30 days of life and the use of antibiotics during the first 30 days of life. All analyses were performed on an intention to treat basis. For all statistical analyses, a two-sided p value of <0·05 was considered significant. For multiple comparisons, a Bonferroni correction was used. SPSS 18·0 (SPSS Inc., Chicago, IL, USA) was used for data analysis.

## Results

**Figure 1:**
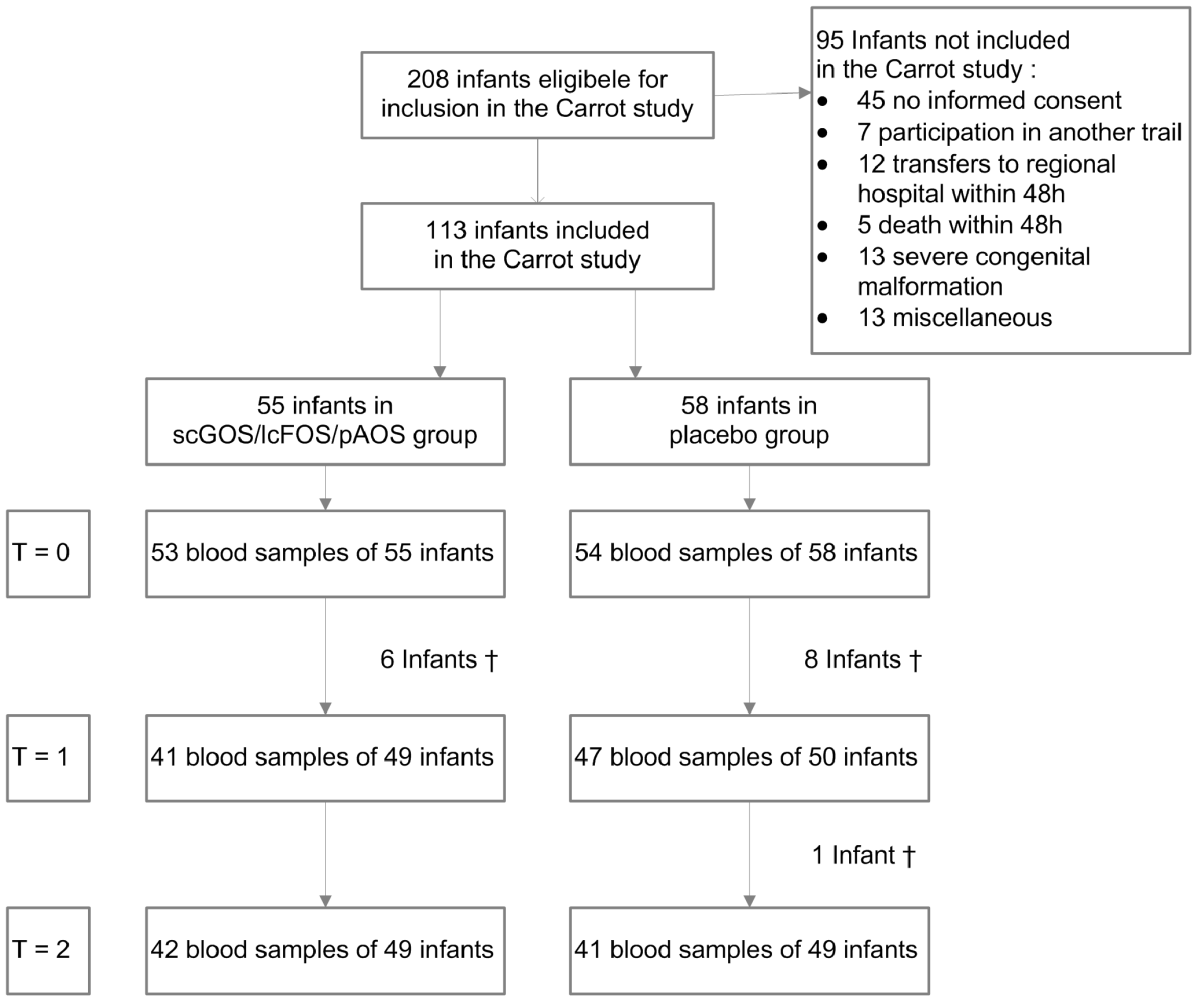
Trial profile. ^†^died.

Between May 2007 and November 2008, 113 preterm infants entered the study (figure 1). Baseline patient and nutritional characteristics were not different in the prebiotic (n = 55) and placebo group (n = 58) (table 1). In total, data of 89% (88/99) of the eligible infants at 5 months and 85% (83/98) of the eligible infants at 12 months of age were collected (figure 1).

**Table 1:** Baseline and nutritional characteristics.

	Prebiotic mixturen = 55			Placebon = 58			
**Baseline characteristics**							
Chorioamnionitis	11	(20%)			13	(22%)	
PE,E or HELLP	17	(31%)			18	(31%)	
Placental insufficiency	4	(7%)			3	(5%)	
Antenatal antibiotics (%)	11	(20%)			16	(28%)	
Antenatal corticosteroids (%)	31	(56%)			32	(56%)	
Multiple birth (%)	9	(16%)			13	(22%)	
Vaginal delivery (%)	31	(56%)			328	(55%)	
Gestational age (wks)	29.9	(1.9)			29.3	(2.1)	
Birth weight (kg)	1.3	(0.4)			1.2	(0.3)	
Birth weight <10th percentile (%)	12	(22%)			8	(14%)	
Sex, male (%)	31	(56%)			36	(62%)	
Apgar at 5 min <6 (%)	9	(16%)			5	(9%)	
Antibiotics postpartum (%)	41	(75%)			44	(76%)	
**Nutritional characteristics**							
Age at start of study supplementation (d)	2.1	(1.5–5.3)			2.1	(1.5–3.3)	
Time to full supplementation dose (d)	11	(4–28)			11	(5–27)	
Mean supplementation dose during study period (g/kg/d)	1.30	(0.1–1.6)			1.27	(0.2–1.8)	
Age at advancement of enteral nutrition (d)	2.8	(0.6–27.5)			2.5	(0.3–18.0	
Exclusive breast milk during 30 day study period (%)	38	(69%)			33	(57%)	

PE, preeclampsia; E, eclampsia; HELLP, syndrome of hemolysis, elevated liver enzymes and low platelets;

PIVH, periventricular-intraventricular hemorrhage.

There were no statistically differences (p<0.05) between both groups.

### scGOS/lcFOS/pAOS Supplementation

**Figure 2:**
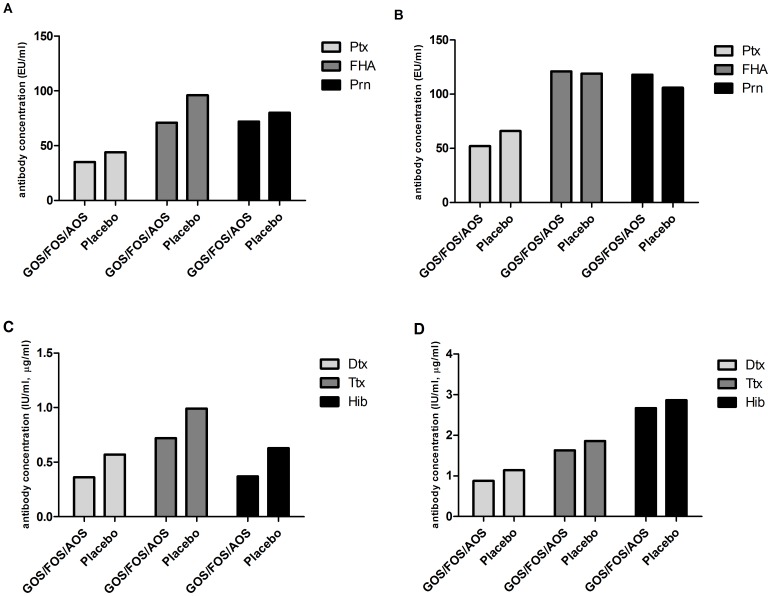
Antibody concentrations preterm infants. **A** Pertussis (Ptx, FHA and PRN) antibody concentrations in at 5 months of age **B** Pertussis (Ptx, FHA and PRN) antibody concentrations at 12 months of age **C** Diphtheria, Tetanus and Hib antibody concentrations 5 months of age **D** Diphtheria, Tetanus and Hib antibody concentrations 12 months of age.

At 5 months of age, enteral supplementation of scGOS/lcFOS/pAOS during the first 30 days of life had no effect on the response on immunizations compared to placebo supplementation. The GMCs of Ptx (37/44 EU/ml), FHA (78/96 EU/ml), Prn (78/80 EU/ml), Dtx (0.40/0.57 IU/ml), Ttx (0.74/0.99 IU/ml) and Hib (0.35/0.63 µg/ml) antibody levels were not different (all p>0.05) in both groups (figure 2 A and 2 C). At 12 months of age, enteral supplementation of scGOS/lcFOS/pAOS had also no effect on the response on immunizations compared to placebo supplementation. The GMCs of Ptx (55/66 EU/ml), FHA (122/119 EU/ml), Prn (116/106 Eu/ml), Dtx (0.88/1.11 IU/ml), Ttx (1.64/1.79 IU/ml) and Hib (2.91/2.55 ug/ml) antibody levels were not different (all p>0.05) in both groups (figure 2 B and 2 D).

### Levels of Protection

Protective antibody concentrations at 5 months and 12 months in the scGOS/lcFOS/pAOS and placebo group combined are shown in table 2. The percentages of preterm infants with protective antibody concentrations for diphtheria and tetanus were both 100%. The percentages of preterm infants with protective antibody concentrations for Hib (64%) and Pertussis toxin (85%) were lower at 5 months compared to diphtheria and tetanus. At 12 months, these percentages increased to 73% for Hib and 95% for Pertussis toxin, however still 27% of the preterm infants did not reach the protective levels for Hib and 5% of the preterm infants did not reach the protective levels for Pertussis.

**Table 2:** Levels of protection in preterm infants.

		Infants with protective concentration (%)	
	International cut off	5 months	12 months
**Pertussis (Ptx)**	≥20 EU/ml	85	95
**Diphtheria (Dtx)**	≥0.01 IU/ml	99	100
**Tetanus (Ttx)**	≥0.01 IU/ml	100	100
**Hib**	≥1 µg/ml	64	73

### Other Influences

Infants with higher BW had higher antibody concentration of Ttx at 5 months,(p = 0.005 ) and higher antibody concentrations of Prn at 5 and 12 months (p = 0.005 and p = 0.008, respectively). Gestational age, breast milk feeding, serious neonatal infections during the first 30 days of life and cord blood antibody concentration had no influence on the antibody concentrations at 5 and 12 months (p>0.05).

## Discussion

Enteral supplementation of a prebiotic mixture consisting of neutral and acidic oligosaccharides during day 3–30 days of life in preterm infants does not enhance the immunization response against diphtheria, tetanus, pertussis and Hib after the primary series immunizations at 5 months nor after the booster immunization at 12 months. No adverse side effects or effects out of the normal range regarding antibody responses have been reported, which supports that the supplementation of scGOS/lcFos/pAOS is safe regarding the immune response to vaccinations.

The infants in this randomized controlled trial received the prebiotic mixture during the first 30 days of life, since the first month after birth is an important period in the evolution of the immune system. Singhal et al. emphasize that early nutrition gives long-term health effects and the first month of life is a critical window. [11] Positive effects on the immune system of preterm infants of feeding with pAOS have been shown, since they are able to act as receptors-analogs and are known to inhibit the adhesion of pathogens on the epithelial surface. [15] In our initial study, we found lower rates of infection in preterm infants supplemented during day 3–30 of life with the prebiotic mixture. [28] We hypothesize that supplementation of scGOS/lcFOS/pAOS during the critical window of the first month diminishes the infection rate by a direct influence on the immune system. By improvement of the immune system, we expected an elevated vaccine response on immunizations. In addition in a mice model, Benyacoub et al. showed that fructo-oligosaccharides (FOS) supplemented to the enteral feeding increased their vaccine response to an oral Salmonella vaccine. Mice receiving FOS during one week before immunisation showed higher IgG levels against Salmonella than mice in the control group. [24] Part of the difference might be explained by the route of vaccination; an oral vaccine in the Salmonella study versus an intramuscular vaccination in our study, or by the timing of oligosaccharides supplementation; one week before vaccination versus day 3–30 of life.

Previously, Westerbeek et al. [36] described the effect of this scGOS/lcFOS/pAOS mixture on the faecal microbiota in preterm infants during day 3–30 of life. At day 14 of life an increase in total bacterial count in the scGOS/lcFOS/pAOS group has been shown, but this effect disappeared at day 30. In addition, a decreased stool pH and a trend towards increased Bifidobacteria have been found. Antibiotics decreased total bacteria count. As preterm infants, with immature immune systems, venous and arterial catheters, long-term intravenous feeding and an overall need for intensive care treatment, have an increased risk for developing infections, they often receive broad-spectrum antibiotics. In our study population, 75% of the infants received antibiotics during the first 30 days of life. Antibiotics and the scGOS/lcFOS/pAOS mixture have counteractive effects on the intestinal microbiota as the scGOS/lcFOS/pAOS mixture is developed to stimulate the normal development of the intestinal microbiota, especially growth of so-called health promoting bacteria, whereas antibiotics inhibit the normal development of the intestinal microbiota, by reducing the number of bacteria, including numbers of bifidobacteria. [37] Antibiotics also alter the composition of the intestinal microbiota and might affect the T cell function. Dethlefsen et al [38] showed a decreased diversity of bacterial strains after the use of oral cyproxin in adults, as several bacterial taxa did not recover after the antibacterial treatment. This alteration of the composition can persist following a short course of antibiotics. [38], [39] Therefore, the scGOS/lcFOS/pAOS mixture and antibiotics have, at least in part, opposite effects on the development of the immature immune system. [36] We hypothesize that frequent use of broad-spectrum antibiotics in our NICU, which was the case in 75% of our study-infants, could have reduced the effect of the scGOS/lcFOS/pAOS mixture on the intestinal microbiota. This might reduce the bacterial growth during the first 30 days of life as described by Westerbeek et al, [36] masking a possible effect of growth of Bifidobacteria bacteria or other health promoting bacteria. The less evident difference in health promoting bacteria growth might in turn lead to a less pronounced positive effect on the immune system and consequently no positive effect on the response to immunizations.

Preterm infants are vaccinated with DTaP-IPV-Hib at 2, 3, 4 and 12 months of uncorrected age, similar to term infants. However, preterm infants have a more immature immune system than term infants, which can impair the response on immunizations, giving lower antibody concentrations after the vaccinations and a lower protection rate.

In total, 27% of the preterm infants vaccinated following the Dutch national immunization schedule did not reach the international assigned protective antibody level for Hib after their booster vaccination at 12 months. This means that they are still vulnerable for Hib infection after a completed immunization schedule. Berrington et al. [5] also found lower percentages of seroprotection for Hib, but in their study an immunization schedule at 2, 4, and 6 months of uncorrected age was used. In 51% of the preterm infants, the Hib antibody concentration was >1 µg/ml after 3 immunizations (at 2, 4, and 6 months). Of the infants with an antibody level <1 µg/ml after 3 immunizations, 93% reached this level after a fourth booster immunization, which is higher than in our study. The different schedule of immunizations with 2 months intervals and last booster immunization at a later age could improve the immune response in preterm infants, but more information about the influence of the timing of vaccinations in preterm infants is needed.

There are some limitations to our study. In a recent study by Prymula et al. [40] prophylactic administration of paracetamol has been shown to impair the immunization response. In our study, we did not collect information about the number of infants who received paracetamol prophylactically before or during immunization.

As preterm infants in our study had lower protective concentrations for Hib after their booster vaccine at 12 months, it is important to study the effects of different immunization schedules in preterm infants.

## Conclusion

Short term enteral supplementation of a prebiotic mixture consisting of scGOS/lcFOS/pAOS during day 3–30 of life does not improve the immunization response in preterm infants, but does not show adverse effects.

## Supporting Information

Checklist S1 **CONSORT Checklist.**
(DOC)

Protocol S1 **Trial Protocol.**
(DOC)
