# Supplementary material for: Neutral and Acidic Oligosaccharides Supplementation Does Not Increase the Vaccine Antibody Response in Preterm Infants in a Randomized Clinical Trial
Source: PLoS One. 2013 Aug 8;8(8):e70904. doi: 10.1371/journal.pone.0070904 (PMC3738516; doi:10.1371/journal.pone.0070904)
Supplement: Protocol S1 — Trial Protocol. (DOC) [file pone.0070904.s002.doc]

Immune effects of acidic and neutral oligosaccharides in the nutrition of preterm Infants: CARROT study.

Investigators:

Elisabeth AM Westerbeek^1^

Ruurd M. van Elburg^1^

Willem P.F. Fetter^1^

Gunther Boehm^2^

Harrie N. Lafeber^1^

**Statistics:**

Jos W.R. Twisk^3^

^1^ Department of Paediatrics, Division of Neonatology, VU University Medical Center, Amsterdam, the Netherlands.

^2^ Numico Research, Friedrichsdorf, Germany.

^3^ Institute of Research in Extramural Medicine, VU University Medical Center,

Amsterdam, the Netherlands.

**Advisors:**

Dept of Pediatrics, VU University Medical Center, Amsterdam: Anemone van den Berg, (Division of Neonatology), A. Marcelien van Furth, (Division of Infectious Diseases), R. Jeroen Vermeulen, (Division of Pediatric Neurology), and John J. Roord.

Dept of Pediatrics, Sophia Children’s Hospital, Rotterdam: Edward E.S. Nieuwenhuis, (Division of Pediatric Gastroenterology and Immunology), Henriette A. Moll*.*

Numico Research, Wageningen: Jan Knol, (Dept of Biomedical Research), Annemiek C. Goedhart (Dept of Project Planning and Management).

National Institute for Public Health and the Environment, Bilthoven: Susan Hahné and Nicolien van der Maas, (Centre for infectious disease control).

# Background

Preterm infants are at increased risk for the development of serious nosocomial infections, especially very low birth weight (VLBW) infants at a NICU.^1^ In a recent review of the literature, we found that the intestinal bacterial colonisation in preterm infants is much more diverse than in term infants and that antibiotics cause a significant delay in the intestinal bacterial colonisation.^2^ Breast milk has a bifidogenic effect on the intestinal bacterial flora.^3,4^ Furthermore, breastfed infants, especially term infants, have less infections and develop less atopy.^5,6^ Many factors have been implicated in this effect, including prebiotics. Addition of prebiotics to infant formula has been found to show potential advantageous effects in term and preterm infants.^7,8^ In breast milk, several types of oligosaccharides can be distinguished: acidic oligosaccharides (20%) and neutral oligosaccharides (80%). In the past, research has mainly focussed on neutral oligosaccharides such as galacto-oligosaccharides and fructo-oligosaccharides (GOS/FOS). As breastfeeding may have anti-inflammatory effects and is thought to prevent the development of atopy, the same potential effects have been attributed to prebiotics. So far, most studies have focussed on GOS/FOS supplementation. Supplementation of GOS/FOS results in:^9,10^

1. stimulation of a bifidogenic flora
2. reduction of pathogens in the intestine
3. production of beneficial fermentation metabolites such as short chain fatty acids (SCFA)
4. decrease of stool pH
5. improved intestinal physiology (stool characteristics, motility)

However, in breastfeeding only 80% of the oligosaccharides are neutral (as in GOS/FOS), whereas 20% are acidic. Acidic oligosaccharides (AOS) can be derived from carrots with their active component pectin. Pectin is a common structural component of all higher plants. Cooking of pectin-containing vegetables induces the cleavage of the long-chain pectin polymers into oligosaccharides. For already nearly 100 years, carrots are known to have health promoting effects. In 1908, carrot soup was used as treatment of diarrhoea.^11^ In 1997, Guggenbichler identified the anti-adhesive effect of acidic oligosaccharides.^12^

In preliminary studies, the combination of acidic and neutral oligosaccharides is more effective than neutral oligosaccharides alone. Preliminary studies indicate that supplementation of acidic oligosaccharides may have several advantageous effects.^13^

1. stimulating a bifidogenic flora
2. preventing adhesion of pathogens to epithelial tissues
3. stimulation of Th1 cytokine response (e.g. TNF-α, IFN-gamma) and decreasing the Th2 cytokine release (e.g. IL-10, IL-4, IL-5)
4. improvement of the response to immunizations

As a result of these effects, it is thought that infants receiving a combination of GOS/FOS with AOS will have:

1. less infections
2. less feeding intolerance
3. less atopy later in life

As infections are still a major cause of morbidity and mortality, reducing the incidence of serious infections is very important. As controversy exist on the definitions for serious infections in neonates, we redefined in a previous study the CDC criteria for serious infections in neonates^1^ and found these criteria applicable in a study in preterm infants.^14^

In the current study, we want to determine the effect of enteral supplementation of acidic and neutral oligosaccharides on the incidence of serious infections, using the previously described criteria for serious infections in preterm infants at high risk for serious infections, with a gestational age < 32 weeks and/or a birthweight < 1500g.

In conclusion, this double-blind randomised controlled trial has the following aims:

1. To determine the effect of acidic and neutral oligosaccharides supplemented enteral nutrition on infectious morbidity, intestinal microflora, feeding tolerance, and short-term outcome in VLBW infants.
2. To elucidate the role of acidic oligosaccharides in modulation of the immune response and postnatal adaptation of the gut.
3. To determine the long term effects of acidic and neutral oligosaccharides supplemented enteral nutrition in the neonatal period on the development of allergic and infectious disease in the first years of life (by questionnaire) and neurodevelopmental outcome, neuromotor development at the corrected age of 1 and 2 years and mental/motor development (BSIDII) at the corrected age of 2 years (as part of the regular follow-up of NICU infants).
4. To determine the effect of acidic and neutral oligosaccharides supplemented enteral nutrition on the response to immunizations with Diphteria-Tetanus-acellular pertussis - inactivated polio - *Haemophilus influenzae* type B – Pneumococcus (DTaP-IPV-HIB-Pneu) vaccination (initial 3 doses), and booster vaccination (4^th^ dose). In infants with parents from endemic Hepatitis B parts of the world and in infants born to Hepatitis B positive mothers, the response to immunization with Hepatitis B vaccin will also be evaluated.

**Methods**

The study is designed as a double-blind randomised clinical trial. Approval of the study protocol by the medical ethical review board of VU University Medical Center Amsterdam will be obtained before the start of the study.

## Study population

Infants with a gestational age <32 weeks and/or birth weight <1500 g admitted to the level III neonatal intensive care unit (NICU) of the VU University Medical Center, Amsterdam, are eligible for participation in the study. Written informed consent is obtained from all parents.

Exclusion criteria are: major congenital or chromosomal anomalies, death <48 h after birth, transfer to another hospital <48 h after birth and admission from an extraregional hospital.

## Treatment allocation and blinding

To balance birth weight distribution into treatment groups, each infant is stratified to one of three birth weight groups (<799 g, 800-1199 g, ≥1200 g) and randomly allocated to treatment <48 hours after birth. An independent researcher uses a computer-generated randomisation table based on blocks of four (provided by Nutricia Nederland BV, Zoetermeer, The Netherlands) to assign infants to treatment A or B, which correspond to batch numbers on the nutrition products. Investigators, parents, medical and nursing staff are unaware of treatment allocation. The code for the batch numbers is broken after data analysis is performed.

**Treatment**

Acidic and neutral oligosaccharides powder and the control powder (maltodextrin) are prepared by Numico Research (Friedrichsdorf Germany) and are sterile packed. During the study period, acidic and neutral oligosaccharides and control powder are monitored for stability and microbiological contamination.

Between days 3 and 30 of life, acidic and neutral oligosaccharides supplementation (20%/80% mixture) is administered in a dose of maximal 1.5g/kg/day to breastfeeding or preterm formula in the intervention group. Two members of the nursing staff daily add supplementation to breast milk or to preterm formula (Nenatal Start®, Nutricia Nederland B.V., Zoetermeer, The Netherlands), according to the parents’ choice. Per 100 ml, Nenatal Start® provides 80 kcal, 2.4g protein (casein-whey protein ratio 40:60), 4.4g fat and 7.8g carbohydrate. When infants are transferred to other hospitals before the end of the study, the protocol is continued under supervision of the principal investigator (IW).

## Nutritional support

Protocol guidelines for the introduction of parenteral and enteral nutrition follow current practice at our NICU. Administration of parenteral nutrition starts at day 1 and will be advanced gradually until amino-acid intake reaches 3 g/kg/day at day 6. Parenteral nutrition is discontinued when enteral feeding reaches a volume of approximately 150 mL/kg/day or less in case of fluid restriction. Parenteral nutrition, an all-in-one mixture provided by the hospital pharmacy, contains per 100 mL 54 kcal, 8.5 g glucose, 1.7 g amino acids and 1.7 g lipids. If necessary, glucose, amino acids and lipids will be given in separate solutions. Full enteral feeding is defined as >120mLkg/day enteral feeding as some infants have fluid restriction.

Guidelines for the introduction of enteral nutrition are as follows: 1. Minimal enteral nutrition (breastfeeding or preterm formula (Nenatal Start®) starts at day 1 (6-12 mL daily); 2. Enteral nutrition is advanced either from day 2 or from day 3-4 in case of: BW <p10, GA <26 weeks, Apgar score at 5 minutes <6, pH <7.10 and/or base deficit >10 mmol/L in arterial cord blood; 3. Feeding is advanced at a dose of 15-20 mL/kg/day to a maximum of 150 mL/kg/day (based on actual weight). Furthermore, guidelines for reduction/withholding of enteral feeding are: 1. Enteral feeding is reduced/withheld in case of gastric residuals (> total volume of past 2 feedings), bilious residuals, emesis, ileus or necrotising enterocolitis Bell’s stage ≥II;^15^ 2. When signs of feeding intolerance resolve, feeding is advanced in the volume given before reduction/withholding within 2 days.

For each infant in the study a feeding schedule is proposed, based on birth weight and the guidelines as mentioned above. However, the medical staff of our NICU has final responsibility for the administration of parenteral nutrition and advancement of enteral feeding.

**Study outcome measures**

##### Study outcome measures

Primary outcome of the study is the effect of acidic and neutral oligosaccharides (20%/80% mixture) supplemented enteral feeding in a maximum doze of 1.5g/kg/day on infectious morbidity as previously defined.^1,14^ Furthermore, parameters of feeding tolerance, and short-term outcome are evaluated (Table 1). In addition to clinical outcome, intestinal permeability, faecal flora (including pH, viscosity measurements and acetate measurements), faecal IL-8 and calprotectin (Diagnostic Products Corporation Nederland BV), gastrointestinal transport time (by carmine red) and plasma cytokine concentrations (Il-2, Il-4, IL-5, IL-8, IL-10, TGFα , IFNγ,) are determined during the 30 day study period (Table 2).

*Clinical outcome measures*

The following perinatal characteristics are registered to assess prognostic similarity: maternal age and race, obstetric diagnosis, administration of antenatal steroids and antibiotics, mode of delivery, sex, gestational age, birth weight, birth weight <p10, Apgar scores, pH of the umbilical artery, clinical risk index for babies^16^ and administration of surfactant.

During the study period actual intake of enteral and parenteral nutrition, powder supplementation and type of feeding (breast milk or preterm formula) are recorded daily.

The occurrence of serious infections is determined by two investigators, unaware of treatment allocation, as previously described.^1,14^ Serious infections include sepsis, pneumonia and meningitis. In addition, the presence of pyelonephritis, and arthritis are recorded. Sepsis work-up consists of blood, cerebrospinal fluid and urine (suprapubic bladder tap) culture. Sepsis is defined as the combination of a positive blood culture and the presence of at least two clinical signs (body temperature <36.5°C or >37.5°C, hypotension, tachycardia, apnoeic attacks, feeding problems, irritability or apathy). Meningitis is diagnosed when micro-organisms are cultured in the cerebrospinal fluid. Pyelonephritis is diagnosed when both urine culture and dimercaptosuccinic acid (DMSA) renal scan are positive. Pneumonia is defined as the combination of a positive culture of tracheal aspirate, bronchial secretion or sputum and the presence of at least one clinical sign in ventilated infants (purulent sputum, changed sputum characteristics or deterioration of ventilation settings) or at least two clinical signs in non-ventilated infants (tachypnea, cyanosis, wheezing/rales/crepitations or purulent sputum/changed sputum characteristics). Arthritis is defined as the combination of a positive culture of intra-articular fluid and the presence of signs of articular inflammation.

*Postnatal adaptation of the gut*

The effect of acidic and neutral oligosaccharides supplemented enteral nutrition on postnatal adaptation of the gut is studied by measuring intestinal permeability and by determining intestinal microflora.

Intestinal permeability is measured by the sugar absorption test, as previously described.^17^ After instillation of the test solution, 2 ml/kg by nasogastric tube, urine is collected for 6 hours. After collection, 0.1 ml chlorohexidine digluconate 20% (preservative) is added to the urine and samples are stored at -20^o^C until analysis. Lactulose and mannitol concentrations (mmol/mol creatinine) are measured by gas chromatography as previously described.^18^ The lactulose/mannitol ratio is calculated and used as a measure of intestinal permeability.

Faecal samples are stored at -20^o^C until analysis by fluorescent in situ hybridisation (FISH) using specific 16S rDNA-targeted probes as described by Harmsen et al.^3^ gastrointestinal transit time (GGT) will be measured by adding 20 mg carmine red to 1 feeding. GGT is defined as the time from feeding the marker to its appearance in the diaper.^19^ Stool pH will be measured using a Handy lab pH meter.^20^

*Immune response*

The effect of acidic and neutral oligosaccharide supplemented enteral nutrition on the immune response is investigated by determining the development of the immune response to Diphteria - Tetanus – acellular Pertussis - inactivated Polio-Haemophilus influenza type B - Pneumococcus (DTaP-IPV-HiB-Pneu) immunizations (first 3 doses), and if indicated Hepatitis B vaccin, and the development of the memory function of the immune response to these immunizations by measuring the response to the 4^th^ booster dose. In addition, the plasma cytokine concentrations (Il-2, Il-4, IL-5, Il-8, IL-10, TGF α, IFN γ), faecal calprotectin and IL-8 concentrations will be measured.

*Follow-up*

To investigate neurodevelopmental outcome, neuromotor development at the corrected age of 1 and 2 years^21^ and mental/motor development (BSIDII) at the corrected age of 2 years are assessed.^22^

To determine the incidence of allergic and infectious disease in the first year of life, faecal samples (FISH, calprotectin and IL-8) and IgE/IgG4 levels will be measured at 5 and 12 months and questionnaires will be sent to the parents prior to the follow-up visit at the corrected age of one year.

To determine the frequency of side-effects of immunizations, questionnaires will be given to the parents at the time of immunizations.^23^

**Sample size**

Based on the differences (76% and 50% respectively) in infectious morbidity in the GEEF study,^14^ and a two-tailed α=0.05, β=0.20, a sample size of 2 x [2*7.85*0.63(0,37)]/(0,26)^2^ = 2 x 54 infants was calculated.

**Statistical analysis**

To determine whether randomisation is successful, prognostic similarity (perinatal and nutritional characteristics) between treatment groups is assessed. The Students’ t-test, Mann-Whitney U test, and chi-square test or Fisher’s exact test are used to compare continuous normally distributed, nonparametric continuous and dichotomous data respectively.

Logistic regression is performed to examine whether acidic and neutral oligosaccharide supplemented enteral nutrition decreases the incidence of serious infections. In an additional analysis, adjustments are made for possible confounding factors as administration of antenatal corticosteroids, birth weight <p10, administration of breast milk and other prognostic factors that may be different between treatment groups.

Analyses of secondary outcomes (only crude) is performed by Mann-Whitney U test, chi-square test or Fisher’s exact test and log rank test for nonparametric continuous, dichotomous data and time-dependent data respectively.

Generalised estimated equations for longitudinal analysis^24^ are used to analyse changes over time in intestinal permeability, faecal microflora, faecal IL-8 and calprotectin, and plasma Th1/Th2 cytokine concentrations.

Distribution of optimal and non-optimal neuromotor development and normal and abnormal mental/motor development in oligosaccharides and control groups is examined by logistic regression with adjustments for possible confounding factors as gestational age and birth weight.

All statistical analyses are performed on an intention to treat basis. In addition, alternative per protocol analyses are performed, excluding all patients who are not treated according to protocol, defined as more than 3 consecutive days or a total of 5 days on minimal enteral feeding or without supplementation. For all statistic analyses a p value <0.05 is considered significant (two-tailed). SPSS 12.0 (SPSS Inc., Chicago, IL, USA) and STAT 7.0 (StatCorp LP, College Station, TX, USA) are used for data analysis.

## Summary

## Background

Prevention of serious infections in very low birth weight (VLBW) infants is a challenge, since prematurity and low birth weight often requires many interventions and high utility of devices. Furthermore, administration of enteral nutrition is limited by immaturity of the gastrointestinal tract. The acid and neutral oligosaccharides play an important role in the development of the intestinal bacterial colonisation and functional integrity of the gut. In preterm infants, the intestinal bacterial colonisation is delayed compared with term infants with possible negative effects on susceptibility to serious infections. An abnormal intestinal bacterial colonisation may also have negative effects on functional integrity of the gut and may lead to impaired function of the immune system. This double-blind randomised controlled trial is designed to investigate the effect of acidic and neutral oligosaccharides supplemented enteral nutrition on infectious morbidity, feeding tolerance and short-term outcome in VLBW infants. Furthermore, an attempt is made to elucidate the role of acidic oligosaccharide supplemented enteral nutrition in postnatal adaptation of the gut and modulation of the immune response.

### Methods

VLBW infants (gestational age <32 weeks and/or birth weight <1500 g) are randomly allocated to receive 1,5g/kg enteral acidic and neutral oligosaccharides (20%/80% mixture) supplementation or placebo (maltodextrin) supplementation between day 3 and 30 of life. Primary outcome is infectious morbidity (defined as the incidence of serious infections). Furthermore, feeding tolerance (defined as a feeding volume ≥120 mL/kg/day), short-term outcome and follow-up are evaluated. The effect of enteral acidic and neutral oligosaccharides supplementation on postnatal adaptation of the gut is investigated by measuring intestinal permeability and determining faecal microflora. The role of acidic and neutral oligosaccharides in modulation of the immune response is investigated by determining immune response to DtaP-IPV-HiB immunizations, plasma cytokine concentrations, faecal calprotectin and IL-8.

**References**

**1.** Van der Zwet WC, Kaiser AM, van Elburg RM, Fetter WPF, Vandenbroucke-Grauls CMJE. Nosocomial infections in a Dutch neonatal intensive care unit: surveillance study with definitions for infection specifically adapted for neonates. J Hosp Infect. 2005;61:300-311.

**2.** [Westerbeek EA](http://www.ncbi.nlm.nih.gov/entrez/query.fcgi?db=pubmed&cmd=Search&itool=pubmed_Abstract&term="Westerbeek+EA"%5BAuthor%5D)M, [van den Berg A](http://www.ncbi.nlm.nih.gov/entrez/query.fcgi?db=pubmed&cmd=Search&itool=pubmed_Abstract&term="van+den+Berg+A"%5BAuthor%5D), [Lafeber HN](http://www.ncbi.nlm.nih.gov/entrez/query.fcgi?db=pubmed&cmd=Search&itool=pubmed_Abstract&term="Lafeber+HN"%5BAuthor%5D), [Knol J](http://www.ncbi.nlm.nih.gov/entrez/query.fcgi?db=pubmed&cmd=Search&itool=pubmed_Abstract&term="Knol+J"%5BAuthor%5D), [Fetter WP](http://www.ncbi.nlm.nih.gov/entrez/query.fcgi?db=pubmed&cmd=Search&itool=pubmed_Abstract&term="Fetter+WP"%5BAuthor%5D)F, [van Elburg RM](http://www.ncbi.nlm.nih.gov/entrez/query.fcgi?db=pubmed&cmd=Search&itool=pubmed_Abstract&term="van+Elburg+RM"%5BAuthor%5D). The intestinal bacterial colonisation in preterm infants: A review of the literature. Clin Nutr. 2006;25:361-368.

**3.** Harmsen HJ, Wildeboer-Veloo AC, Raangs GC, Wagendorp AA, Klijn N, Bindels JG, Welling GW. Analysis of intestinal flora development in breast-fed and formula-fed infants by using identification and detection methods. J Pediatr Gastroenterol Nutr 2000;30:61-67

**4.** Yoshioka H, Iseki K, Fuijta K. Development and differences of intestinal flora in the neonatal period in vreast-fed and bottle-fed infants. Pediatr 1983;72:317-321.

**5.** Holt PG, Sly PD, Bjorksten B. Atopic versus infectious diseases in childhood: a question of balance? Pediatr Allergy Immunol 1997;8:53-58.

**6.** Kull I, Wickman M, Lilja G, Nordvall SL, Pershagen G. Breast feeding and allergic diseases in infants-a prospective birth cohort study. Arch Dis Child 2002;87:478-481.

**7.** [Fanaro S](http://www.ncbi.nlm.nih.gov/entrez/query.fcgi?db=pubmed&cmd=Search&itool=pubmed_Abstract&term="Fanaro+S"%5BAuthor%5D), [Boehm G](http://www.ncbi.nlm.nih.gov/entrez/query.fcgi?db=pubmed&cmd=Search&itool=pubmed_Abstract&term="Boehm+G"%5BAuthor%5D), [Garssen J](http://www.ncbi.nlm.nih.gov/entrez/query.fcgi?db=pubmed&cmd=Search&itool=pubmed_Abstract&term="Garssen+J"%5BAuthor%5D), [Knol J](http://www.ncbi.nlm.nih.gov/entrez/query.fcgi?db=pubmed&cmd=Search&itool=pubmed_Abstract&term="Knol+J"%5BAuthor%5D), [Mosca F](http://www.ncbi.nlm.nih.gov/entrez/query.fcgi?db=pubmed&cmd=Search&itool=pubmed_Abstract&term="Mosca+F"%5BAuthor%5D), [Stahl B](http://www.ncbi.nlm.nih.gov/entrez/query.fcgi?db=pubmed&cmd=Search&itool=pubmed_Abstract&term="Stahl+B"%5BAuthor%5D), [Vigi V](http://www.ncbi.nlm.nih.gov/entrez/query.fcgi?db=pubmed&cmd=Search&itool=pubmed_Abstract&term="Vigi+V"%5BAuthor%5D).Galacto-oligosaccharides and long-chain fructo-oligosaccharides as prebiotics in infant formulas: a review. Acta Paediatr Suppl. 2005;94:22-26.

**8.** [Boehm G, Jelinek J, Stahl B, van Laere K, Knol J, Fanaro S, Moro G, Vigi V.](http://www.ncbi.nlm.nih.gov/entrez/query.fcgi?cmd=Retrieve&db=pubmed&dopt=Abstract&list_uids=15220664&query_hl=6&itool=pubmed_docsum) Prebiotics in infant formulas. J Clin Gastroenterol. 2004;38:S76-79.

**9.** Boehm G, Lidestri M, Casetta P, Jelinek J, et al. Supplementation of a bovine milk formula with an oligo-saccharide mixture increases counts of faecal bifidobacteria in preterm infants. Arch Dis Child Fetal Neonatal Ed 2002;86:F178-81.

**10**. Knol J, Boehm G, Lidestri M, Negretti F, Jelinek J, Agosti M, Stahl B, Marini A, Mosca F. Increase of faecal bifidobacteria due to dietary oligosaccharides induces a reduction of clinically relevant pathogen germs in the faeces of formula-fed pretern infants. Acta Paediatr 2005;94;31-33.

**11**. Moro E. Karottensuppe bei ernährungsstörungen der säuglinge. München Med Wschr 1908;31:1637-1640.

**12**. Guggenbichler JP, De Bettignies-Dutz A, Meissner P, Schellmoser S, Jurentsch J. Acidic oligosaccharides from natural sources bloch adgerence of Escherichia coli on uroepithelial cells. Pharm Pharmacol Lett 1997;7:35-38.

**13.** Boehm G, Stahl B. Oligosaccharides. In: Mattila-Sandholm T, ed. Functional Dairy Products. Cambridge: Woodhead, 2002: p 203-243.

**14.** van den Berg A, van Elburg RM, Westerbeek EAM, Twisk JWR, Fetter WPF. Glutamine-enriched enteral nutrition in very-low-birth-weight infants and effects on feeding tolerance and infectious morbidity: a randomized controlled trial. Am J Clin Nutr 2005;81:1397-1404.

**15.** Bell MJ, Ternberg JL, Feigin RD et al. Neonatal necrotizing enterocolitis. Therapeutic decisions based upon clinical staging. Ann Surg 1978;187:1-7.

**16.** The CRIB (clinical risk index for babies) score: a tool for assessing initial neonatal risk and comparing performance of neonatal intensive care units. The International Neonatal Network.
Lancet. 1993;342:193-198.

**17.** van Elburg RM, Fetter WP, Bunkers CM, Heymans HS. Intestinal permeability in relation to birth weight and gestational and postnatal age. Arch Dis Child Fetal Neonatal Ed 2003;88:F52-F55.

**18.** van Elburg RM, Uil JJ, Kokke FT, Mulder AM, van de Broek WG, Mulder CJ, Heymans HS. Repeatability of the sugar-absorption test, using lactulose and mannitol, for measuring intestinal permeability for sugars.J Pediatr Gastroenterol Nutr. 1995;20:18418-8.

**19.** Mihatsch WA, Högel J, Pohlandt F. Hydrolyzed protein accelerates the gastro-intestinal transport of formula in preterm infants. Acta Paediatr 2001;90:196-8.

**20.** Fanaro S, Jelinek J, Stahl B, Boehm G, Kock R, Vigi V. Acidic oligosaccharides from pectin hydrolysate as new component for infant formulae: Effect on intestinal flora stool characteristics, and pH. J Pediatr Gastroenterol Nutr. 2005;41:186-190.

**21.** Samsom JF, de Groot L: The influence of postural control on motility and hand function in a group of 'high risk' preterm infants at 1 year of age. Early Hum Dev 2000;60:101-113.

**22.** Bayley N. Bayley Scales of Infant development II 2nd ed. 1993. New York, The Psychological Corporation.

**23**. Veiligheidsbewaking en consultatie RVP, Centrum infectieziektenbestrijding. RIVM,

Bilthoven.

**24.** Twisk J.W.R.: *Applied longitudinal data analysis for epidemiology. A practical guide.* Cambridge UK: Cambridge University Press; 2003.

**25.** Usher R, McLean F. Intrauterine growth of live-born Caucasian infants at sea level: standards obtained from measurements in 7 dimensions of infants born between 25 and 44 weeks of gestation. J Pediatr 1969;74:901-10

**26.** Jobe AH, Bancalari E. Bronchopulmonary dyplasia. Am J Respir Crit Care Med 2001;163:1723-9

**27.** Papile LA, Burstein J, Burstein R, Koffler H. Incidence and evolution of subependymal and intraventricular hemorrhage: a study of infants with birth weights less than 1,500 gm. J Pediatr 1978;92:529-34.

**28.** An international classification of retinopathy of prematurity. The committee for the Clasification of Retinopathy of Prematurity. Arch Ophthalmol 1984;102:1130-4.

Appendix 1: Case record form

Appendix 2: Questionnaire side effects vaccinations

**Tables**

### Table 1 Clinical outcome measures

|  |  | Remarks |
| --- | --- | --- |
| Infectious morbidity |  |  |
| Serious infections |  | Primary outcome |
| Number of infectious episodes |  |  |
| Cultured micro-organisms |  |  |
| Feeding tolerance |  |  |
| Enteral feeding >120 mL/kg/day |  |  |
| Age at finishing parenteral nutrition |  |  |
| Necrotising enterocolitis |  | Bell et al.[15] |
| Weight *z* scores at birth, day 30 and at discharge |  | Usher et al. [25] |
| Patent ductus arteriosus |  |  |
| Ventilatory support |  |  |
| Use of oxygen at postmenstrual age of 36 weeks |  | Jobe et al. [26] |
| Intraventricular hemorrhage |  | Papile et al. [27] |
| Retinopathy of prematurity |  | Committee for ROP [28] |
| Death |  |  |
| Age at discharge from NICU and at discharge home |  |  |
| ROP= retinopathy of prematurity; NICU = neonatal intensive care unit. | | |

Table 2: Study schedule

| **Postnatal age** |  | **< 48h** | **day 4** | **day 7** | **day 14** | **day 30** | **5 mo** | **1 yr** |
| --- | --- | --- | --- | --- | --- | --- | --- | --- |
| **Faeces** |  |  |  |  |  |  |  |  |
| Faecal  Calprotectin  /IL-8 |  | x | - | x | x | x | x | x |
| Intestinal  microflora  FISH / pH |  | x | - | x | x | x | x | x |
| Gastrointesti-  nal transport  time |  | - | - | - | x | - | - | - |
| **Urine** |  |  |  |  |  |  |  |  |
| Intestinal  permeability |  | x | x | x | - | - | - | - |
| **Blood** |  |  |  |  |  |  |  |  |
| Cytokine profile | 0,5ml | x | - | x | x | - | - | - |
| IgE/IgG4 | 0,6ml | - | - | - | - | - | x | x |
| Immune  response | 2,0ml | - | - | - | - | - | x | x |
